# An overview of image-based phenotyping as an adaptive 4.0 technology for studying plant abiotic stress: A bibliometric and literature review

**DOI:** 10.1016/j.heliyon.2023.e21650

**Published:** 2023-11-02

**Authors:** Muhammad Fuad Anshori, Andi Dirpan, Trias Sitaresmi, Riccardo Rossi, Muh Farid, Aris Hairmansis, Bambang Sapta Purwoko, Willy Bayuardi Suwarno, Yudhistira Nugraha

**Affiliations:** a Department of Agronomy, Hasanuddin University, Makassar, 90245, Indonesia; b Department of Agricultural Technology, Hasanuddin University, Makassar, 90245, Indonesia; c Center of Excellence in Science and Technology on Food Product Diversification, 90245, Makassar, Indonesia; d Research Center for Food Crops, Research Organization for Agriculture and Food, National Research and Innovation Agency, 16911, Cibinong, Indonesia; e Department of Agriculture, Food, Environment and Forestry (DAGRI), University of Florence (UNIFI), Piazzale delle Cascine 18, 50144, Florence, Italy; f Department of Agronomy and Horticulture, Faculty of Agriculture, IPB University, Bogor, 11680, Indonesia

**Keywords:** Analytic review, Digitalization 4.0 technology, High-throughput phenotyping, Plant breeding, Salinity stress, Water deficit

## Abstract

Improving the tolerance of crop species to abiotic stresses that limit plant growth and productivity is essential for mitigating the emerging problems of global warming. In this context, imaged data analysis represents an effective method in the 4.0 technology era, where this method has the non-destructive and recursive characterization of plant phenotypic traits as selection criteria. So, the plant breeders are helped in the development of adapted and climate-resilient crop varieties. Although image-based phenotyping has recently resulted in remarkable improvements for identifying the crop status under a range of growing conditions, the topic of its application for assessing the plant behavioral responses to abiotic stressors has not yet been extensively reviewed. For such a purpose, bibliometric analysis is an ideal analytical concept to analyze the evolution and interplay of image-based phenotyping to abiotic stresses by objectively reviewing the literature in light of existing database. Bibliometricy, a bibliometric analysis was applied using a systematic methodology which involved data mining, mining data improvement and analysis, and manuscript construction. The obtained results indicate that there are 554 documents related to image-based phenotyping to abiotic stress until 5 January 2023. All document showed the future development trends of image-based phenotyping will be mainly centered in the United States, European continent and China. The keywords analysis major focus to the application of 4.0 technology and machine learning in plant breeding, especially to create the tolerant variety under abiotic stresses. Drought and saline become an abiotic stress often using image-based phenotyping. Besides that, the rice, wheat and maize as the main commodities in this topic. In conclusion, the present work provides information on resolutive interactions in developing image-based phenotyping to abiotic stress, especially optimizing high-throughput sensors in image-based phenotyping for the future development.

## 1 Introduction

Achieving food security is a concern of all nations and it has been implemented in the agreement of sustainable development goal (SDG). The world now facing increasing population growth, that always correlates with the increasing demand for food needs [[1], [2], [3]]. It is therefore very important to increase food production, distribution and access worldwide. However, this effort meets with the global warming that exacerbating the food crisis especially in the developing countries [4,5]. On the other hand, the conversion of arable land to non-agricultural propose has been increased drastically and reduced productive land for food [[6], [7], [8]]. Shifting agriculture production to unfavorable lands is become a necessitate options to unsure food supply [[9], [10], [11]]. The unfavorable land means there are many stresses (biotic and abiotic) during plant growth that affect the production [3,10]. Scientists try to develop variety and cultivation technology that can cope with many kinds of environmental stresses [[12], [13], [14]]. Hence, it is important to develop and apply adaptive technologies in the food sector that are beneficial for the farmers.

Image-based phenotyping (IBP) is an adaptive technology that utilizes the development of digitalization 4.0 technology. This technology is very useful in increasing the precision of assessment or evaluation of plants in various environments, including stress environments [[15], [16], [17]]. Conventional phenotyping is commonly used in plant tolerance screening as it results in relatively easy-to-perform and inexpensive destructive samplings. However, the success of this approach is dependent on population size of breeding material and technical skill of breeders [16,18]. Direct selection in physiological response is more accurate in identifying desirable targeted plants, but this method needs sophisticated tool for measurement biochemical or substance that immerse during stresses. In some case the physiological parameter identification needs to destroy the plant which mean there will be impossible to generate new offspring or new population. Meanwhile indirect selection using molecular or DNA markers still considerable high cost and depend on the close linkage between markers and targeted gene, as well as the heritability of the markers [[19], [20], [21]]. Both approaches have a high level of accuracy and precision; thus, underestimates or overestimates during selection step can be avoided [16,22]. However, these two approaches are highly complex and relatively expensive [22,23]. Therefore, IBP is a new approach for plant evaluation and identification of morphology and physiological response to a single plant or population under abiotic stress tolerance.

Most obvious implementation of IBP is in the field of plant breeding. Plant breeding programs have a limited set of resources to phenotype of breeding population [[24], [25], [26], [27]]. The population size can determine the success of breeding program, the higher population the better of chance to get desirable candidate variety. However, at the same time large population requests more resources should be allocated [26,27]. Therefore, the IBP is crucial to have robust and accurate selection tools to assist plant breeders in screening their breeding material.

The IBP approach have widely used in recent years for the plant breeding program and physiological analysis of several plant species, including rice [16,28,29], potato [30], corn [31,32], wheat [33,34], arabidopsis [35]. In general, plant phenotype is the accumulation and collaboration of various interrelated genes; thus, conventional plant phenotype assessment cannot collect interactions and associations of these genes. In addition, the use of image processing characteristics is correlated with physiological traits [15,17,36] and genetics [37]. This approach can morphologically, physiologically, and genetically explain the characteristics of a population. It indicates that IBP can address these limitations of human vision [35,[38], [39], [40]], including screening plant tolerance to abiotic stress. However, information on developments, trends, challenges, and opportunities of imaged-based phenotyping to abiotic stress has not been widely reported. Therefore, a database-based system must be identified through bibliometrics and literature review.

Bibliometric is a concept of literature review, which is based on comprehensive information from a collection of articles on a specific aspect [[41], [42], [43], [44]]. This concept is compelling in identifying research developments and trends, which serves as the basis for strategies in developing a precise science [41,45,46]. This bibliometric concept provides many benefits in mapping concepts to specific research topics. Some key elements in bibliometrics include number, trend, and interaction, whether based on country, publisher, or author, as well as the development of topics that become strategic issues in an area of study [41,42,45,47]. The bibliometric concept will help the literature review become more focused. Such elements lead to the development of this literature concept in the last decade, including the agricultural sector [[48], [49], [50], [51], [52], [53]]. Costa et al. and Kolhar and Jagtap reported bibliometric analysis of plant or IBP [45,54]. However, both articles do not focus on abiotic stress studies. In addition, these two bibliometrics only explain general developments and interactions that occur, but do not explain in depth the image-based phenotypic description, either through the concept of literature review or systematic review. Therefore, IBP to abiotic stresses can be further studied through bibliometric analysis and literature review.

This study aims to investigate the development of articles in general on the topic of IBP to abiotic stresses and the trend globally among countries. We explored the topic development based on keywords interactions on this topic, furthermore we discussed the general concept development of IBP in related with abiotic stress on plant science.

## 2 Materials and methods

**Fig. 1:**
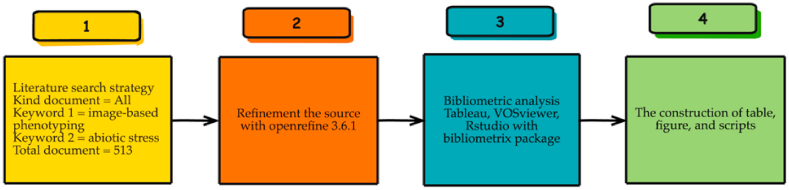
Scheme of the bibliometric approach used to review the topic of IBP to abiotic stresses.

This study used a systematic methodology in which the concept of bibliometric analysis of image-based phenotyping (IBP) to abiotic stresses has four main stages (Fig. 1). The initial step involved data mining which was conducted through subject-specific literature search. The second phase entailed improving Scopus data mining [45,48,52,55]. The third phase consists of bibliometric analysis based on multiple parameters. The final step involved the development of the results. Meanwhile, the literature review only used the related references with the focus keywords that result from bibliometric analysis. The bibliometric methodology applied for complete each step is detailed described in the following sub-sections.

### 2.1 Literature search strategy

The literature is derived from document mining on Scopus. On January 5, 2023, bibliographic data mining was conducted. Scopus centered its data mining efforts on two search categories with the concept of all sources and endless time. The first search field is IBP, and the second search field is abiotic stress or codenamed ALL (image-based AND phenotyping) AND ALL (abiotic AND stress). The range of collecting data is less than 5 January 2023. Combining the two search fields resulted in 554 documents related to IBP to abiotic stresses. The documents obtained from Scopus data mining include 482 journals, 41 books, 20 Book Series, 10 conference proceedings, and one trade journal.

Meanwhile, the data mined includes citations, bibliographical information, abstract, keywords, and other data (trade names and manufacturers, accession numbers and chemicals, conference information, and references). The citations include author name, author Scopus ID, document title, year, source title, volume, issue, page, citation count, source and document type, and publishing stage. The bibliography includes affiliation and publisher. The abstract and keywords include author keywords and index keywords.

### 2.2 Refinement of the search results

Mining data from Scopus was enhanced using OpenRefine 3.6.1 (BSD 2-Clause License) and JAVA. This software will eliminate errors and irrelevant articles from mined data files. The process is focused on the Author and index keywords in the mined data. The data in both categories are split and grouped against words with the same meaning or purpose. After that, the keywords that have been grouped are combined back into one. It also improves the accuracy of Scopus data mining results. Therefore, the notion of mining data refining and filtering is performed semi-manually.

### 2.3 Bibliometric analysis

There are five approaches to bibliometric analysis: general information, global topic development, and topic development based on keywords. The analysis uses three types of software, namely, Tableau, VOSviewer, and Rstudio (version 3.6.1, R Studio Inc., Boston, MA, USA), with the bibliometrix package. Tableau is the widely used visualization software. This software can summarize the advantages of various software such as GIS, FRED, Infographics, or Excel. Consequently, the data analysis sector highly values this program [56]. In this study, Tableau only analyzes the number of documents per country. VOSviewer focuses on document data and relational knowledge units for document construction [57]. VOSviewer creates scientific knowledge maps that show the relationship among the literature on this topic. This software has large-scale graphical presentation and multifunctional adaptability to source data from different database formats [42](. The application of this software focuses on clusters of interaction and collaboration across countries, interactions among keywords, and citation interactions among publishers. Other analyses use Rstudio and the bibliometrix package. This package can fit the bibliometric concept, and it is free. Furthermore, this software may collect a variety of data mining sources, improve reference disambiguation through string-based algorithms, implement direct and tri-cited analysis, and use a hybrid method that combines bibli-ometric and semantic approaches. The last development includes the detection of term explosions by thematic mapping, which can smooth topics and evolution, and latent semantic analysis [58].

## 3 Results and discussion

### 3.1 Expansion of the subject of IBP to abiotic

The 554 documents obtained from Scopus containing some generic information. From 2010 to 2023, 251 sources were used to construct this topic's database with 64,097 references, 2765 writers and 1566 author's keywords are identified. In addition, 16 documents are written by a single author. This topic also has 37 % international co-authorship and 6.46 co-authors per document. Moreover, the average number of citations per paper on this subject is 28.85.

**Fig. 2:**
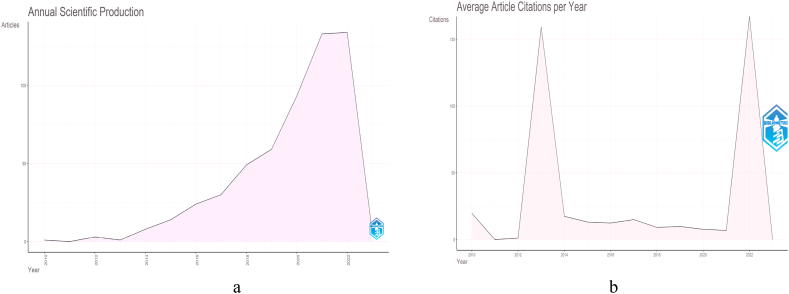
Development of documents related to IBP to abiotic stress studies: (a) the number of documents produced per year and (b) the trend of citations per year.

The development of the number of documents and citations which become a fundamental topic in bibliographic analysis is presented in Fig. 2. These two pieces of information become the starting point for analyzing the importance of an issue in the future. The number of documents produced on image-based phenotyping (IBP) focusing abiotic stresses has drastically increased since 2013 (Fig. 2a). The sharpest increase occurred from 2019 to 2021. However, the increase has been sloping from 2021 to 2022. This kind of decrease it is probably related to the global COVID-19 Emergency which has prevented the normal performance of research activities, especially in a field such as that of phenotyping for the analysis of plant responses to abiotic stresses which require continuous data acquisition over time. Meanwhile, the highest number of citations was recorded in 2013 and 2022 (Fig. 2b). Based on document result analysis, IBP for abiotic stress has become crucial in recent years. The number of publications still has an increasing trend with high citation in 2022. It is in line with dynamic climate change [59]. So, a systematic and straightforward approach is needed to detect physiological mechanisms and plant tolerance to these stresses, like the IBP concept. According to Costa et al. [45], plant phenotyping has more rapid trends than other approaches in plant evaluation and has a strong linkage to precision agriculture. Therefore, the topic of IBP to abiotic stresses has good momentum in its development, particularly at the end of the citation momentum occurring in 2022.

### 3.2 Development of the topic of IBP to abiotic stresses globally across countries

**Fig. 3:**
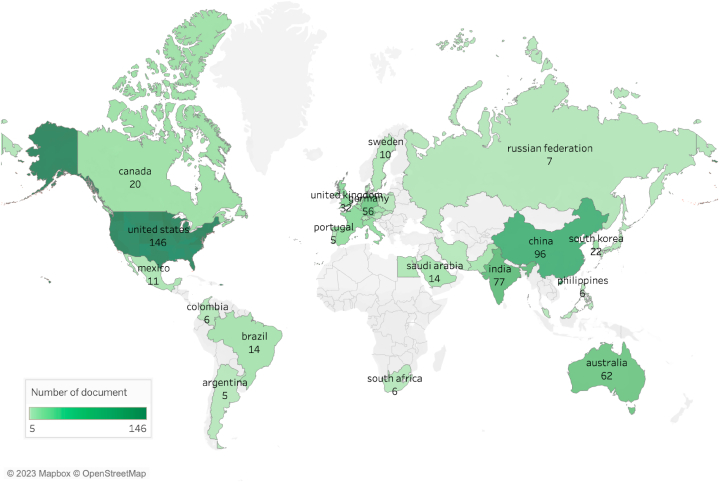
Countries that have published the most articles on image-based phenotyping under abiotic stresses.

The global IBP to abiotic stresses can be seen from the number of documents per country, the trend of document development in each country, corresponding issues across countries, and co-responding interactions among countries. In general, the product of papers related to IBP to abiotic stresses is evenly distributed on the European continent. By contrast, the lowest development associated with this topic document is found on the African continent. However, when analyzed by country, the top five countries with the highest number of documents related to the topic are scattered across various continents, apart from the African continent, including the European continent (282 documents, especially in Germany (59 documents), United States (155 documents), China (103 documents), India (90 documents), and Australia (64 documents); Fig. 3 and Supplementary 1). Based on the production of papers per year, the beginning of the development of this topic occurred in 2010. These results follow the reports of Zhang et al. [42], Costa et al. [45], Kolhar and Jagtap [54], who state that the United States and China are the largest countries for the development of precision plant phenotyping. However, in this review, China shows a drastic increase in slope every year, particularly from 2020 to 2022.

**Fig. 4:**
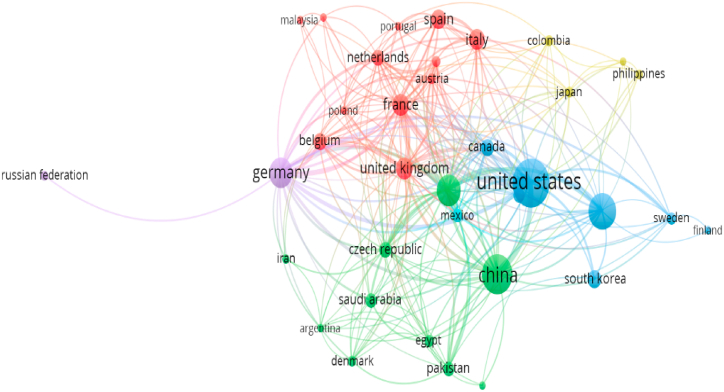
Interaction and collaboration clusters across nations concerning image-based phenotyping under abiotic stresses.

**Fig. 5:**
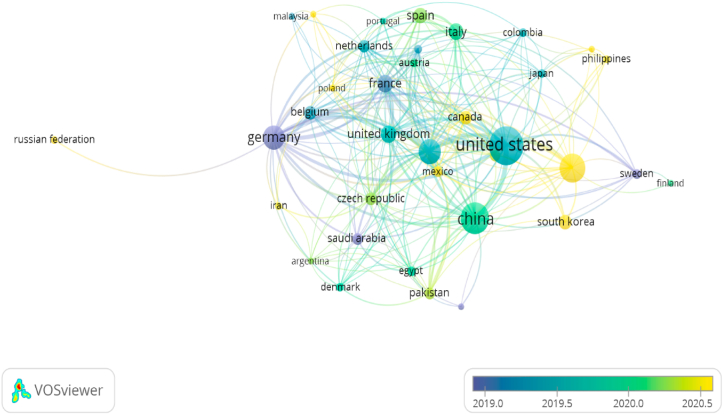
Clusters of international interaction and collaboration on image-based phenotyping under abiotic stresses based on time.

Based on interactions among countries, there were five main clusters (Fig. 4). The first cluster is centered in Germany (purple in Figure 4). This country is the first to initiate the development of the topic of IBP to abiotic stresses (Fig. 5). It is also a line with Zhang et al. [42] focused on Agriculture multispectral technology. The concept of IBP is significantly dependent on image sensor technology. It indicated the initial idea could be learning in Germany. Then, the development extends to other European countries as the second cluster (Fig. 5). This cluster is centered in France, which consists of the Netherland, Belgium, Poland, Spain, Italy, and Switzerland (red cluster in Fig. 4). This result also stated by Costa et al. [45] that United Europe (EU) had the most significant publication number on prior 2019. However, after 2019, the United States (US) production increased dramatically. The United States is the focal point of the third cluster (blues cluster in Fig. 4). In general, the US interacts with other countries in its cluster, such as Australia, Canada, Mexico, and South Korea, which were only developed in over 2020 (Fig. 5). Nevertheless, the US article production is significantly more than in other countries except China. China has grown after the US cluster but not ahead of Australia and South Korea. The China cluster consists of Iran, Argentina, Denmark, the Czech Republic, Saudi Arabia, Egypt, Pakistan, and Slovakia (Fig. 5). Based on all country clusters, the topic of IBP to abiotic stress centered in UE in prior 2019 year. Then, this concept rapidly comprehensive to other countries, especially massive population countries like the USA, China (1412.3 million), and India (139.3 million). It also fits with the countries’ rising populations (332.3 million; Statista 2023). Consequently, they have a considerable quantity of publications on this subject.

### 3.3 Development of keywords on IBP to abiotic stresses

**Fig. 6:**
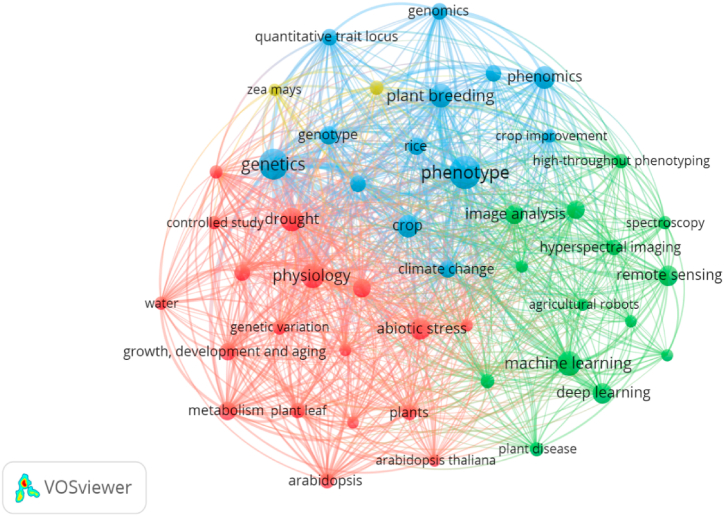
Keywords Interaction related to IBP to abiotic stresses.

The results of keyword interaction analysis use an occurrence limit of 15, so there are 72 appropriate keywords. After that, the keywords are filtered and produce 51 that interact with each other. The results of the interaction of these keywords show four color groups: blue, red, green, and yellow (Fig. 6). However, the yellow group is minor and only consists of “Zea mays” and “biomass”.

The blue group focuses on phenotype keywords as the main keywords of the blue group and is also the main center in analyzing these keywords. Apart from the phenotype, the dominant keywords in the blue cluster are “genetics”, “genotype”, “crop”, “plant breeding”, “phenomics”, “wheat”, “rice”, “quantitative trait locus”, and “genomics”. All members of the keywords in this group are closely related to the direction of using the IBP method in abiotic stress studies. In general, this concept benefits plant breeding programs, especially in the phenotyping or phenomics of a plant line [ [26,60,61]]. A close interaction between plant breeding keywords with phenotype and phenomics also characterizes it. The conventional phenotyping approach to plant breeding needs to improve its accuracy so that IBP can improve its selection accuracy [ [60,61]]. In addition, this cluster also explains that phenotype and plant breeding are closely related to genetic concepts, such as genomics and quantitative trait locus (QTL). Plant breeding is closely associated with genetic traits passed down from generation to generation, so the development of phenotyping with precision will involve genetic factors [[61], [62], [63]]. Several studies have carried out this concept, particularly in developing QTLs and genome-wide association studies on a character [[64], [65], [66]]. Meanwhile, the keywords rice, and wheat show that the use of the concept of IBP to abiotic stress for breeding purposes is widely practiced in these two commodities. This was also reported by Kim et al. [ [34]]. The articles related to the two commodities also follow the great demand for these commodities as the world's staple food [67].

The red clusters focus on physiology, drought, and metabolism. Other members of this cluster consist of “genetic variation”, “adaptation”, “controlled study”, “arabidopsis”, “plant roots”, “plants”, “chlorophyll”, and “growth, development, and aging”. Based on all keyword members, this group focuses on physiological aspects, types, and stress parameters used in the IBP concept of abiotic stress. For example, giving stress to plants will disrupt plant physiological and metabolic processes so that plants will produce specific symptoms in response to this stress [[68], [69], [70]]. These response symptoms can be detected through an IBP approach so that several IBP studies will validate the results of this analysis on physiological and metabolic parameters [[71], [72], [73]].

**Fig. 7:**
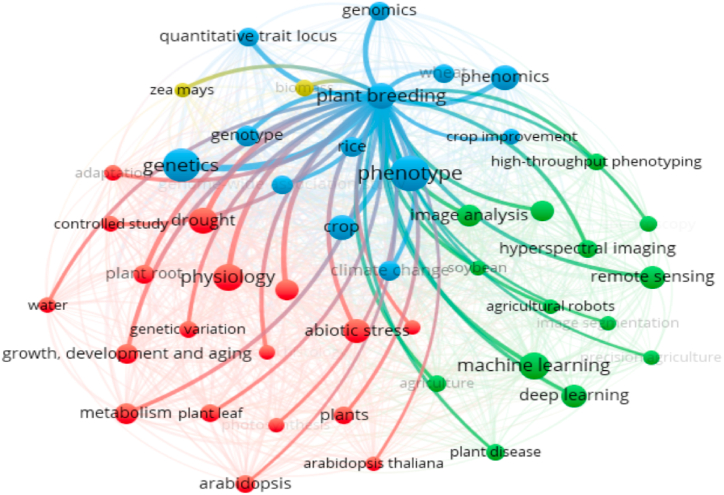
The interaction of “plant breeding” keywords to others keywords.

The dominant stresses that are often detected with this concept are drought and salinity. The joining of drought and salinity tolerance in this cluster reflects this. Drought and salinity generally have the same response, namely water deficit. Water plays a critical role in plant physiological processes, so a lack of water will inhibit and damage existing metabolic processes [[74], [75], [76], [77]]. There are two visible effects and symptoms when experiencing a water deficit: the chlorophyll content of leaves and the development of plant roots. Water deficit will cause photosynthesis to be hampered, so the photosynthate produced is not balanced with the photosynthesis consumed. This will cause oxidative stress, damaging the leaf chlorophyll, so the leaves experience senescence [78]. Low photosynthate produced, and water content will affect cell division so that plant development will be hampered [8,75,[79], [80], [81]]. The roots, as the part that interacts directly with water, will show the most development growth due to progressive soil drying [75,82]. This underlies the chlorophyll content of leaves and root development as crucial aspects of plant growth in water deficit stress. Therefore, IBP in plant breeding must be validated by observing plant physiology and growth, namely chlorophyll, photosynthesis, plant root, and growth, development, and aging. This is also reflected in the interactions that occur between the concepts of physiology and plant breeding in Fig. 7.

The last large cluster (green) has a lot of keyword interaction centers, namely high-throughput phenotyping, remote sensing, deep learning, machine learning, and image analysis. In addition, other cluster members consist of “agricultural robots”, “agriculture”, “image segmentation”, “hyperspectral imaging”, and “plant disease”. In general, this cluster is a method of development of IBP. When viewed from many keywords, this group of keywords develops concurrently. The concept of high-throughput phenotyping is a trend carried out in IBP [60,72,[83], [84], [85], [86], [87]]. In general, the HTP is a nondestructive and rapid approach of continuously monitoring and measuring multiple phenotypic traits of multiple plants related to the growth, yield and adaptation to biotic or abiotic stresses. This concept uses sensor digitization combined with automation principles to increase the accuracy of these observations, especially in assessing plant productivity, pigment content, tolerance, and disease [[85], [86], [87]]. This is the basis for why image segmentation, hyperspectral imaging, and plant disease are included in this cluster. In addition, the concept of high-throughput phenotyping is further enriched by the acquisition, processing and analysis of big data, namely remote sensing, deep learning, and machine learning [84,85] (Jayasinghe et al., 2020; Jangra et al., 2021). Remote sensing the science and technology of obtaining reliable information about physical objects (i.e., crops/plants) and the environment through the acquisition, processing and interpreting of data sensed from space (e.g., satellite-based) or sky (e.g., aircraft- and drone-based) [[83], [84], [85],^87^]. Remote sensing technologies allow the acquisition of time-series data over large areas in a short period of time, making it suitable for field applications. Therefore, using high-throughput phenotyping technology based on big data (machine and deep learning) analysis will make it easier for plant breeders to assemble a variety, including in supporting climate change research (Fig. 7).

**Fig. 8:**
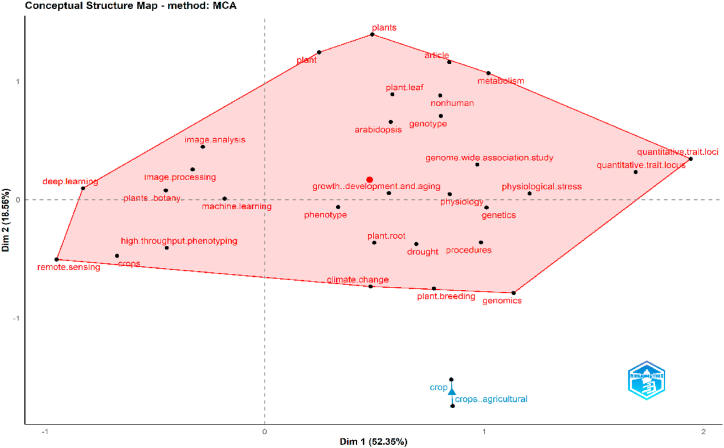
Conceptual framework for IBP to abiotic stresses.

These keywords can also be clustered with the concept of multiple correspondence analysis as a conceptual structure analysis (Fig. 8). The result shows two clusters. The keywords “plant”, “metabolism”, “remote sensing”, “quantitative trait loci”, “plant breeding”, “genomics”, “climate change”, and “deep learning” are the keywords with the most incredible diversity in the central cluster (colored red, Fig. 8). By contrast, the second cluster only consists of two keywords: “crop” and “crop agricultural”. Conceptual structure analysis focuses on the outermost point of the cluster. This point is the highest diversity of the dimensional combinations of a variance partition, so the outer point in this analysis can be a conceptual reference for future research progress [58]. Therefore, the results highlight that in future it will be important to apply deep learning methods for analysing remotely sensed data in order to relate the genotypic (e.g., metabolic) and phenotypic (i.e., morpho-physiology) responses of plants to abiotic stresses. This will be mandatory to obtain genetic gains in plant breeding for more resilient varieties or species.

## 4 Overview of image-based phenotyping to abiotic stresses

The IBP in the topic of 4.0 adaptive technology is the development of precision phenotyping methods to detect the effect of abiotic stresses on the action and symptoms of plant growth comprehensively. This concept can be assess plant potency gradually under stress in each plant phase, especially with colorimetric and segmentation [28,66,83,[88], [89], [90]]. This differs from conventional observations, which only use a few representative characters to achieve a goal [[91], [92], [93]]. Although in some approaches, observations can be made with hand-handling tools and destructive sampling. Both methods have better precision coverage than conventional morphology. However, using this concept requires time, energy, cost, and many samples [66,72,94,95] In plant breeding, each line represents its potential from a large population, so destructive observation of lines becomes ineffective in assessing their optimal potential [60,66,87,96]. Therefore, the plant breeding approach is more directed at the concept of IBP, especially in the topic of stress breeding.

IBP applications are also optimized with High-throughput phenotyping (HTP) technology. HTP relies on computer science, engineering, and big data analysis developments to accelerate plant assessment. Taking pictures is carried out automatically and thoroughly depending on the expected goals, so the observation process becomes efficient with high precision [85,97]. This concept is also widely applied in the identification of tolerance, especially in drought stress [33,34,61,[98], [99], [100]] and salinity [[101], [102], [103], [104]]. However, the effectiveness of the HTP concept is also influenced by several factors, namely the environment of the observation area or platform, the sensors used, and the method used for data processing and analysis [84,85,105].

Generally, there are three platform concepts in shooting: artificial in a growth chamber or controlled environment, field ground-based, and field aerial-based [72,84,86]. The controlled environment is a common and basic platform used in IBP [106]. This platform has a reasonable repetition rate, easy to handle, low bias, and good resolution [72]. This advantage allows the modeling and evaluation process to be stable and directed. The use of the concept of a controlled environment in drought stress and salinity has also been reported by Haimansis et al. [28], Laraswati et al. [16], and Sakinah et al. [93] on rice; Wu et al. [107] on corn; and Kim et al. [34] on wheat. However, controlled environment platforms require high costs, and monitoring has a limited scope. In addition, these platforms are useful in a preliminary research phase but then the results must be tested in the field for greater agronomic significance. This is due to the high diversity of environments in the field, so precision models in a controlled environment may only sometimes be appropriately applied in the field. Meanwhile, the platforms in the field are divided into two platforms, namely ground and aerial-based [33,72,84]. A ground-based platform has the advantages of flexible deployment and good spatial resolution [34,72,84,108]. However, the capturing area still needs to be improved, and it takes a long time to cover the entire planting area. In addition, you could state that several ground-based vehicles can damage the plants during their passage in the inter-row or compact the soil (and therefore compromise the growth of the crop and the experimental results when applied for analysing the abiotic stress responses). In contrast, an aerial-based platform has broad coverage, but its effectiveness depends on the weather condition is an additional limiting factor. Moreover, it would be useful to state that UAV technology is very expensive and requires skilled operators, while most of the satellite images can be downloaded for free from the web [33,84]. The use of field-based concepts in stressful environments was also reported by Kim et al. [34] on wheat; Qiu et al. [31] and Wu et al. [32] on maize; and Jiang et al. [109] on rice. Field-based environments require exact multispectral and hyperspectral sensors [34,84]. This aims to avoid bias due to sunlight, especially in cloudy conditions, so that the capture result can be analyzed in more detail.

Using sensor types and phenotyping methods also influences the effectiveness of HTP in IBP to abiotic stress studies [33,72,83,85] The use of sensors is identical to the capacity to receive light waves. In general, several types of sensor technology are based on these light waves, namely gamma, X-ray, UV, Visible light, near-infrared, and long-wave infrared [72,110]. Visible light is a wavelength that is often and commonly used [60]. The concept of visible light uses red, green, and blue channels (RGB), as the basis for determining the color of each pixel. However, the RGB approach is widely sensitive to electromagnetic radiation so this sensor can have a higher bias than other sensors [72,85]. Several studies have reported the effectiveness of this sensor for abiotic stresses, especially drought and or salinity, namely Haimansis et al. [28], Laraswati et al. [16], Jiang et al. [109], and Sakinah et al. [93] on rice; Munns et al. [111], Paul et al. [112], and Nehe et al. [113] on wheat; and Zhang et al. [114], Qiu et al. [86], Wu et al. [32] and Dodig et al. [115] on maize. Another sensor that can be used in visible light is Light detection and ranging (LIDAR). LIDAR is an RGB sensor that can be used in dark conditions with high resolution. Sharpness, accuracy, and good resolution make this tool often used in constructing phenotyping based on three dimensions (3D) or remote sensing concepts. The basis of the LIDAR sensor is a laser fired to see the density of the canopy and aspects of plant growth. However, this tool is relatively expensive, complex, and requires a long time and complicated analysis [33,72,83,116] The use of this sensor on abiotic stress (drought and/or salinity) has been reported by Su et al. [117] on maize and Aneley et al. [118]on potatoes.

Gamma, X-ray, and UV wavelengths are shorter than visible light [72,83,85]. This makes the energy produced in the three waves huge compared to visible light, especially in gamma rays. This enormous energy allows the light to penetrate parts of the plant so that this light can be used to see the inside of metabolic processes, transport pathways, and ion translocation in plants [119]. Positron emission tomography (PET) is a sensor used at gamma wavelengths [72]. This technology has been used in the animal and human worlds [85,119]. In general, PET can precisely monitor the translocation of nutrients in vivo with high resolution [85,[119], [120], [121]]. This is very effective for visualization under salinity and heavy metal stress [72,121]. In addition, this sensor is not affected by environmental conditions and produces quantitative and visual data [119,121,122]. This facilitates the process of interpretation. The application of this sensor to abiotic stress has been reported by Ariño-Estrada et al. [123] in detecting salinity absorption in green foxtails. However, this sensor analysis requires a large amount of money and has not been widely used for plants, so the development process is still not expansive [85]. The X-ray CT sensor is used at X-ray wavelengths [72,83,85]. This sensor also views morphology and plant tissue parts illustrated in 3D tomographic images [[124], [125], [126], [127]]. This sensor is widely applied to form root architecture found underground [125]. Several applications of this method to abiotic stress have been reported by van Harsselaar et al. [128] on potato drought stress, Schmidt et al. [129] on wheat drought stress, and Kehoe et al. [130] on the detection of barley aerenchyma in submergence stress. Meanwhile, the sensor used at the UV level is Chlorophyll fluorescence (ChF) [34,72,85,131,132]. This sensor will produce false colors that can be used to predict photosynthetic potential, and the health of the canopy or plant leaves against abiotic stress [34,133]. The false color received by the sensor results from the reflection from leaf chlorophyll, indicating the effectiveness of plant photosynthetic phytochemicals [72,134]. The effectiveness of the reflection of chlorophyll can be translated as a parameter of the potential quantum yield of photosystem II (Fv/Fm) [72,133,135,136]. However, using this sensor requires adjustments to measurements in dark conditions or are strongly influenced by observational light conditions [34,85]. Several abiotic stress studies that have used this tool are Phaseela et al. [136] on drought stress and rice salinity; Larouk et al. [137], Sherstneva et al. [138] and Todorova et al. [139] on wheat drought stress; and Kopsell et al. [140] on maize drought stress.

The last category of sensors is those used at a wavelength greater than visible light. This indicates that the energy released at this wavelength is relatively weak. However, the energy provided at these wavelengths can detect surface temperature and biochemical reflections from the tested image [84]. Sensors in this category consist of Near-infrared (NIR)/short-wave infrared (SWIR), hyperspectral imaging, thermal infrared, and magnetic resonance imaging (MRI) [72,84]. NIR/SWIR is a transition sensor from visible light [141]. This sensor will receive reflected radiation from leaf chlorophyll, including leaf reflections transmitted by the canopy from top to bottom, so that architecture, thickness, and leaf moisture content can be detected in this system [83,141,142]. This makes observation non-destructive and easy to do. Several studies have reported the use of this sensor in abiotic stress, namely Phansak et al. [143] and Pabuayon et al. [104] on drought stress and rice salinity; Mokhtari et al. [144], Fan et al. [145], and Danzi et al. [146] on wheat drought; and Casaretto et al. [147] on corn drought. However, this type of sensor is sensitive to wind and cloud cover and requires ground background correction [84]. Hyperspectral sensors are characterized by a wide wavelength range, which guarantees a greater level of accuracy than NIR, especially in detecting plant resistance and tolerance [72,[148], [149], [150], [151], [152]]. For such an example, non-destructive screening of phenotypes within the hyperspectral range allows for quantification of secondary metabolites, including phenolic compounds and flavonoids, involved in plant responses and adaptation to both biotic and abiotic stress conditions [[153], [154], [155]]. The accuracy of this sensor makes them often used in aerial field-based concepts [84,85,149]. However, their weakness, apart from the high cost, is that the interpretation of the results requires more in-depth data analysis [85]. Thermal infrared and MRI sensors fall into the long-wave infrared subcategory. Thermal infrared has a more detailed level than NIR [72]. In general, the higher the sensor's wavelength, the lower the error from the absorption of infrared radiation [85]. This further increases the precision in identifying temperature and water status in the canopy or leaves and stomatal conductivity [[156], [157], [158]]. However, this concept has areas for improvement regarding its impact on the surrounding environment and data reproducibility, requiring a strict and controlled protocol [85,156,157]. Several studies have used thermal imaging sensors for abiotic stress, namely, rice against drought stress [159] and salinity [101,160]; on wheat to drought stress [[161], [162], [163], [164]] and sodic soils [165]. Meanwhile, the MRI sensor moves on radio waves to detect water protons in plant metabolism [72,166]. This sensor uses nuclear magnetic resonance signals from several atomic nuclei (1H, 13C, 14 N, and 15 N) to generate phenotyping images [85,166]. The results from the sensor are based on 3D images. The high sensitivity of this sensor allows the MRI sensor to detect water content, plant health, plant metabolism, and plant nutrient transport [166,167]. However, this sensor also has high costs and low resolution for large cells [85,166]. The sensor concepts are briefly shown in Table 1.

**Table 1:** Summary regarding the use of sensors in the concept of high throughput phenotypes.

Kind of light	Sensor	Wavelength	Phenotype parameter	IBP Parameter	Advantages	Disadvantages	Abiotic stress	Reviewed
Gamma	positron emission tomography (PET)	>10pm	Transport and translocation partitioning, flow velocity, sectorality	region-of-interest (ROI) analysis, color intensity, maximum intensity projection, colour code	can measure in vivo and is not affected by environmental conditions; Provides visual and quantitative information; measure large dimensions	Limited spatial resolution, high cost, and till date limited application in plants	salinity, heavy metal stress and drought	Li et al. [83], Ariño-Estrada et al. [123]; Antonecchia et al. [121]; Mincke et al. [119]; Galieni et al. [122], Jangra et al. [85], Al-Tamimi et al. [72]
X-ray	X-ray CT scan	10nm-10pm	Morphometric parameters in 3D, flow speed, viability and quality of seed, tissue slice	Micro and macro segmentation	Non-invasive and nondestructive 3D images	Non-invasive and nondestructive 3D images	drought, heat, and submergence	Teramoto et al. [125]; Piovesan et al. [126]; van Harsselaar et al. [128], Schmidt et al. [129], Kehoe et al. [130]; Rippner et al. [127].
UV	Chlorophyll fluorescence (ChF)	10 nm–400 nm	Photosynthetic status, architecture, chlorophyll conductance, leaf health status, non-photochemical quenching, pigment composition, and quantum yield	time to reach maximal fluorescence (Tf(max)), Area, F0, FM, VJ, VI, FV, ΦE0, ΦD0, SFI(abs), PI(abs)	Detect stress before the appearance of visual symptoms	require adjustment of measurement in dark conditions or heavily affected by observation light conditions	drought, heat, cold and salinity	Li et al. [83], Faseela et al. [134], Guidi et al. [135], Larouk et al. [137], Jangra et al. [85], Sherstneva et al. [138], Todorova et al. [139], Al-Tamimi et al. [72]
Visible Light	RGB	400–700 nm	Morphology, geometric, seed germination and colorimetric	Colorimetric (Red index, Green index, Blue Index), color area, segmentation, geometric, saturation, convex	Low cost and simple	broad sensitivity to electromagnetic radiation, has low accuracy in predicting plant biochemistry	salinity, heat, heavy metal stress, submergence, nutrient deficiency and drought	Li et al. [83], Zhang et al. [114], Paul et al. [112], Qiu et al. [86], Jayasinghe et al. [84], Wu et al. (2021) [32], Laraswati et al. [16], Jiang et al. [109], Sakinah et al. [93], Jangra et al. [85]
	Light detection and ranging	400–700 nm	RGB images, chlorophyll fluorescence, photochemical reflectance index and leaf temperatures	Colorimetric, geometric, area, segmentation, convex, angle	high data resolution, can be operated at night	vast volumes of data, difficult analysis	drought, heat, nutrient deficiency and salinity	Li et al. [83], Su et al. [117], Kim et al. [178], Jangra et al. [85], Al-Tamimi et al. [72], Aneley et al. [118], Hama et al. [116]
Near infrared	NIR/SWIR	1000–2500 nm	water content composition parameters, leaf area index	NIR-SWIR index, vegetation index (Normalized difference vegetation index (NDVI), extended multiplicative signal correction (EMSC), e.t.c) and colorimetric	Quick and easy; images can be further processed; no damage	Needs soil background corrections; affected by wind and transient cloudiness	nutrient deficiency, drought, heat and salinity	Li et al. [83], Gutiérrez-Rodríguez et al. [142], Casaretto et al. [147], Fan et al. [145], Holzman et al. [141], Jangra et al. [85], Phansak et al. [143], Al-Tamimi et al. [72], Pabuayon et al. [104], Danzi et al. [146]
	hyperspectral imaging	350–2500 nm	Leaf and canopy water status, pigment composition, leaf and canopy health status, plant nutrition, leaf growth, photosynthesis rates, coverage density, stress-related secondary metabolites	Color intensity, vegetation index (NDVI, Normalized difference red edge (NDRE)), Red Green Ratio Index (RGRI), water band index (WBI), Photochemical reflectance index (PRI), Moisture stress index (MSI), Modified chlorophyll absorption in reflective index (MCARI), Modified anthocyanin reflectance index (mARI)	Better prediction accuracy; measurement over a wide range; identification of certain diseases	Sophisticated models making data interpretation difficult; relatively slow; expensive	nutrient deficiency, old, drought, heat and salinity	Li et al. [83], Jayasinghe et al. [84], Alvarez et al. [152]; Mertens et al. [149], Jangra et al. [85], Al-Tamimi et al. [72], Sytar et al. [153], Sytar et al. [154], Jayapal et al. [155]
long-wave infrared (microwave)	thermal infrared	>2500 nm	Leaf area index, plant water status, seed composition, transpiration and stomata conductance, canopy or leaf temperature	normalized relative canopy temperature (NRCT) index, crop water stress index (CWSI), Canopy temperature depression (CTD), colorimetric	The main concern is robustness; reproducibility and data analysis; must follow a strict protocol	highly influenced by environmental factors; reproducibility and data analysis; need to follow strict protocol	nutrient deficiency, drought, salinity	Li et al. [83], Bhandari et al. [162], Das et al. [165], Jangra et al. [85], Mahreen et al. [159], Menegassi et al. [160], Ashfaq et al. [163], Qin et al. [164], Al-Tamimi et al. [72]
	magnetic resonance imaging (MRI)	>2500 nm	Morphometric parameters in 3D, Water status, plant health status, metabolic study, transportation	Morphometric, colourimetric, colour code, geometric, and Segmentation	Simple sample preparation (select cases); fast; whole plants and plant cell cultures as in vivo can be measured	Low resolution in case of giant cells	Drought	Borisjuk et al. ([166]; Pfugfelder et al. [167], Jangra et al. [85], Al-Tamimi et al. [72]

The method selected for analyzing the large amount of acquired data is the last factor influencing HTP in IBP. HTP will produce comprehensive data because it is done automatically and on a large population. This will raise many decision-making considerations [72,85,168]. Suppose the resulting HTP data needs to be analyzed systematically and in-depth. In that case, the final decision of the analysis will be ambiguous, so the analysis requires precise concepts such as machine and deep learning analysis. Both analyses are part of artificial intelligence, which aims to streamline complex populations' automatic and systematic decision-making [72,[168], [169], [170]]. The concept of analysis is carried out by creating a pattern and system from big data and integrating it with specific algorithms into an automated system [[170], [171], [172]]. This is very efficient with computer vision data for plant phenotyping, especially for breeding stress plants that carry out large populations in the straining process [72,169]. This effectiveness increases by combining big data from other approaches, such as genomics, transcriptomics, proteomics, and metabolomics. Consequently, the adoption of multiple technical and conceptual capacities pertaining to IBP has the potential to promote novel breeding programs for speeding up genetic improvements of crop plants under different environments [169].

In this view, several international and regional networking initiatives have recently flourished to effectively integrate existing phenotyping facilities, technologies, data, methodologies, services, and resources [173,174]. As the world's major plant phenotyping hub, the International Plant Phenotyping Network (IPPN; available online at: https://www.plant-phenotyping.org/) aims to foster interaction between academia, industry, policy and general public stakeholders by favoring synergic training activities which are pivotal for advancements in phenotypic data-driven breeding. Under such domain, large investments for plant phenomics research and cross-disciplinary infrastructures have been made in China, Asia, North America and Europe, including China Plant Phenotyping Network (CPPN), Asia-Pacific Plant Phenotyping Conference (APPP, http://www.appp-con.com), North American Plant Phenotyping Network (NAPPN, https://www.plantphenotyping.org) and European Plant Phenotyping Network 2020 (EPPN2020, https://cordis.europa.eu/project/id/731013). Europe is globally recognized as the leader hub in whole plant phenotyping pipeline, providing shared analytic approaches and interoperable frameworks through excellence scientific community [175]. For instance, the European Infrastructure for Multi-Site Plant Phenotyping (EMPHASIS, https://emphasis.plant-phenotyping.eu) aims at developing and implementing pan-European plant phenotyping facilities that can intensify cross-national selection gains of climate-resilient crop plants. Within the EMPHASIS consortium, national networks such as the German Plant Phenotyping Network (DPPN, https://dppn.plant-phenotyping-network.de), the French Plant Phenotyping Network (FPPN, https://www.phenome-emphasis.fr), the Italian Plant Phenotyping Network (PHEN-ITALY, http://www.phen-italy.it) and the Austrian Plant Phenotyping Network (APNN, https://appn.at) have enhanced the adoption of IBP approaches for in-depth comprehension of the complex genotype x environment x management (GxExM) interactions underlying plant adaptation to abiotic stresses [176]. However, a greater international co-ordination is still indispensable to transfer technological and methodological IBP know-how also to emerging countries on which the 4.0 digitalization phenomics have unique potential for overcoming the global food security challenge under unfavorable environmental scenarios [177].

## 5 Conclusion and future perspectives

Based on the results of bibliometric analysis of IBP to abiotic stress, this field is projected to continue to evolve over time. It is based on the fact that the quantity of documents and citations for this issue continues to increase, allowing it to develop sustainably. The center of the development of this topic will remain in two countries, namely, China and the United States. This significant increase in publications of the United States, China, and India to this topic also are based on the increased population of their country. These countries have been categorized as the big three of population in the world (United Nation 2022). The impact of global warming is directly on them, so they must stimulate creating adaptive varieties under abiotic stress. Therefore, the publication about IBP to abiotic stress, particularly in US and China, will increase over time. In addition, several countries such as South Korea, Brazil, and Canada will experience a drastic increase in the relatively large number of articles on this topic, with a new development starting in 2020. These results can also motivate Southeast Asian countries to use this topic as an option in developing publications in the 4.0 era.

The results of bibliometric analysis of keywords show that the development of IBP to abiotic stress is closely related to genetics, genomics, plant breeding, and physiology stress. These words are critical aspects in developing adaptive variety under abiotic stress. Genetics, genomics (i.e., qtl, gwas, and genome editing), and physiology have expensive costs to detect the adaptive variety, so in the future, IBP can help the developing activity in creating a variety based on the relationship at the OMICS level.

Based on abiotic stress kind, drought and saline becomes an abiotic stress often using IBP. Both stresses are closely related to water and oxidative stress. The effect of these stresses can be detected with color contrast and segmentation of plant growth based on IBP. The difference in color contrast and segmentation area is evident in tolerant and sensitive varieties. So, this difference helps to predict the degree of adverse stress of crops. Therefore, the use of IBP is more directed toward drought stress and heat stress.

In future results, IBP to abiotic stress will be related to high-throughput phenotyping (HTP). The HTP is the precision agriculture concept to help the plant breeding program. The effectiveness of HTP is highly dependent on three factors: the platform (i.e., controlled environment and field-based), sensors (i.e., RGB, hyperspectral, thermal, MRI, PET or X-Ray) and distance of the acquiring sensor from the target plant/crop (i.e., proximal and remore sensing), and big data analysis applied. The sensor is crucial as the core of the HTP component. The more precision sensor can develop more parameters in the plant. Meanwhile, using big data analysis will make determining plant tolerance to stress easier based on high-throughput phenotyping (remote sensing and image analysis (2D and 3D)). In general, big data analysis and precision agriculture are the powerful and core approaches in the 4.0 technology era, so the concept of IBP depends on the sensor technology's rhythm and big data analysis development. Therefore, this developmental concept will be integrated into plant breeding; thus, the assembly of varieties becomes more precise.

## Data availability statement

Data will be made available on request.

## Declaration of competing interest

The authors declare that they have no known competing financial interests or personal relationships that could have appeared to influence the work reported in this paper.
