# Supplementary material for: An overview of image-based phenotyping as an adaptive 4.0 technology for studying plant abiotic stress: A bibliometric and literature review
Source: Heliyon. 2023 Nov 2;9(11):e21650. doi: 10.1016/j.heliyon.2023.e21650 (PMC10660044; doi:10.1016/j.heliyon.2023.e21650)
Supplement: Multimedia component 1 [file mmc1.pdf]

Supplementary 1. document information for each country on the topic of image-based phenotypes on abiotic stress studies.

No	label	x	y	cluster	Weight <Links>	Weight <Total link strength>	weight<Documents >	weight<Citations>	weight<Norm. citations>	score<Avg. citations>	score<Avg. norm. citations>
1	argentina	-0.1299	0.8845	2	12	13	5	34	2.5985	6.8	0.5197
2	australia	0.1564	0.0221	3	27	111	62	2969	79.246	47.8871	1.2782
3	austria	-0.0305	-0.3726	3	17	26	8	217	14.806	27.125	1.8508
4	belgium	-0.5213	-0.0431	1	16	40	19	537	16.0986	28.2632	0.8473
5	brazil	0.7067	-0.2482	4	14	19	14	130	13.8698	9.2857	0.9907
6	canada	0.2849	-0.3228	3	18	39	20	228	24.856	11.4	1.2428
7	china	0.483	0.3181	2	24	98	96	2762	142.3518	28.7708	1.4828
8	colombia	-0.0482	-0.5534	1	9	9	6	161	4.5374	26.8333	0.7562
9	czech republic	-0.066	0.4332	2	23	48	18	437	30.6059	24.2778	1.7003
10	denmark	0.0949	0.9103	2	12	18	8	110	5.7239	13.75	0.7155
11	egypt	0.3813	0.7173	2	15	21	10	135	7.166	13.5	0.7166
12	france	-0.2055	-0.1889	1	28	73	33	1031	39.3044	31.2424	1.191
13	germany	-0.8144	0.1822	5	28	115	56	2436	77.3782	43.5	1.3818
14	india	0.7569	-0.0808	3	20	53	77	811	62.8344	10.5325	0.816
15	iran	-0.3486	0.7451	2	4	5	6	56	4.9725	9.3333	0.8287
16	italy	-0.2581	-0.7354	1	13	38	27	479	23.4572	17.7407	0.8688
17	japan	0.4944	-0.3981	3	14	16	8	338	9.8359	42.25	1.2295
18	malaysia	-0.8651	-0.3099	1	5	5	6	182	7.4673	30.3333	1.2446
19	mexico	0.3851	0.0511	3	15	42	11	291	23.6214	26.4545	2.1474
20	netherlands	-0.336	-0.3617	1	19	41	16	671	31.556	41.9375	1.9723
21	pakistan	0.6266	0.665	2	13	30	13	301	16.2764	23.1538	1.252
22	philippines	0.9264	-0.4989	3	5	7	6	253	5.4615	42.1667	0.9102
23	poland	-0.4105	0.0701	1	17	22	5	105	12.2645	21	2.4529
24	portugal	-0.479	-0.4176	1	10	11	5	73	3.3332	14.6	0.6666
25	russian federation	-2.0784	0.4989	5	1	2	7	27	5.0304	3.8571	0.7186
26	saudi arabia	0.029	0.658	2	13	26	14	1143	17.9676	81.6429	1.2834
27	slovakia	0.6735	0.8124	2	4	5	5	81	3.6076	16.2	0.7215
28	south africa	-0.7624	-0.3356	1	8	10	6	20	6.9934	3.3333	1.1656
29	south korea	1.1891	-0.0796	4	4	11	22	312	13.4274	14.1818	0.6103
30	spain	-0.3521	-0.6068	1	15	35	25	393	24.2686	15.72	0.9707
31	sweden	0.0901	-0.8712	5	10	19	10	117	7.2648	11.7	0.7265
32	switzerland	0.0336	-0.4886	3	16	32	8	328	16.3605	41	2.0451
33	united kingdom	-0.1157	0.0728	1	27	80	32	1356	71.7432	42.375	2.242
34	united states	0.5099	-0.1278	4	28	160	146	5067	189.8904	34.7055	1.3006
